# Brain stimulation over dorsomedial prefrontal cortex causally affects metacognitive bias but not mentalising

**DOI:** 10.3758/s13415-025-01277-1

**Published:** 2025-02-26

**Authors:** Rebekka S. Mattes, Alexander Soutschek

**Affiliations:** Department for Psychology, Ludwig-Maximilians-Universität München, Leopoldstr. 13, 80802 Munich, Germany

**Keywords:** Metacognition, Mentalising, DmPFC, TDCS, Metacognitive bias, Metacognitive sensitivity, Metacognitive efficiency

## Abstract

Despite the importance of metacognition for everyday decision-making, its neural substrates are far from understood. Recent neuroimaging studies linked metacognitive processes to dorsomedial prefrontal cortex (dmPFC), a region known to be involved in monitoring task difficulty. dmPFC is also thought to be involved in mentalising, consistent with theoretical accounts of metacognition as a self-directed subform of mentalising. However, it is unclear whether, and if so how, dmPFC causally affects metacognitive judgements, and whether this can be explained by a more general role of dmPFC for mentalising. To test this, participants performed two tasks targeting metacognition in perceptual decisions and mentalising whilst undergoing excitatory anodal versus sham dmPFC tDCS. dmPFC tDCS significantly decreased subjective confidence reports while leaving first-level performance in accuracy and reaction times unaffected, suggesting a causal contribution of dmPFC to representing metacognitive bias. Furthermore, we found no effect of dmPFC tDCS on neither metacognitive sensitivity and efficiency nor on mentalising, providing no evidence for an overlap between perceptual metacognition and mentalising in the dmPFC. Together, our findings highlight the dmPFC’s causal role in metacognition by representing subjective confidence during evaluations of cognitive performance.

## Introduction

Our everyday decisions often come with a degree of uncertainty due to the brain’s limited processing capacity (Fleming, 2021). To manage this uncertainty, the brain employs metacognitive processes, or “thinking about thinking,” which involves assessing and evaluating our levels of uncertainty in decision-making processes (Metcalfe, 1994). Effective metacognition is crucial for strategy selection, error identification, and course correction, optimizing behavioural outcomes and enabling us to navigate complex daily tasks (Yeung & Summerfield, 2012). Deficits in metacognition can severely impact decision-making, rendering its study vital for understanding neuropsychiatric disorders and developing therapeutic interventions (David et al., 2014; Vaccaro & Fleming, 2018). Despite the importance of metacognition, the neural mechanisms causally underlying metacognitive judgements are far from understood. The current study was designed to determine the causal role of the dorsomedial prefrontal cortex (dmPFC) in metacognition.

The dmPFC belongs to a prefrontal brain network that has been associated with metacognitive processes in previous imaging studies (for a meta-analysis, see Vaccaro & Fleming, 2018), including also the frontopolar cortex (FPC) (Fleming et al., 2012), ventromedial PFC (vmPFC) (Schnyer et al., 2005), and dorsolateral PFC (dlPFC) (Rounis et al., 2010). Beyond such correlative evidence, the causal contributions of the dlPFC and FPC to metacognition were already evidenced by past brain stimulation studies (Rahnev et al., 2016; Rounis et al., 2010; Ryals et al., 2016; Shekhar & Rahnev, 2018; Soutschek et al., 2021). Less is known, however, about the causal role of the dmPFC for metacognitive judgements. In a recent fMRI meta-analysis by Vaccaro & Fleming (2018), the dmPFC was found to correlate with metacognition and specifically with levels of self-reported confidence. This is in line with the established functional role of the dmPFC for flexible, adaptive control of behaviour, including error monitoring (Brown & Braver, 2005; Carter et al., 1998), conflict monitoring (Barch et al., 2000; Botvinick et al., 1999; Kerns et al., 2004; Oehrn et al., 2014; Soutschek et al., 2013), and decision-making under uncertainty (Christian et al., 2024; Hadland et al., 2003; Kennerley et al., 2006; Rushworth et al., 2004; for a comprehensive review see Clairis & Lopez-Persem, 2023). The findings from metacognition research suggest that the dmPFC might play a role in evaluating not only objective but also subjective task difficulty and uncertainty. However, it remains unclear whether, and if so how, dmPFC activation causally influences metacognitive judgements.

The dmPFC might contribute to dissociable subfacets of metacognition: metacognitive bias, metacognitive sensitivity, or metacognitive efficiency. If reported postdecision confidence is generally high (irrespective of the accuracy of the decision), this reflects a high metacognitive bias, whereas a low metacognitive bias is characterised by generally low levels of confidence. In contrast, individuals with high metacognitive sensitivity report confidence levels that typically align with their actual performance (i.e., higher confidence after correct relative to incorrect decisions), demonstrating an ability to precisely identify and communicate variations in their objective task performance (Fleming & Lau, 2014). Lastly, metacognitive efficiency captures the metacognitive skills of a person by setting metacognitive sensitivity in relation to first-level task performance. In a recent study, Ting et al. (2023) found a negative correlation between metacognitive bias and dmPFC activity. Similarly, Vaccaro & Fleming (2018) observed a correlative link between medial PFC activity and confidence levels, whereas their results on metacognitive sensitivity remained inconclusive. Hence, the question remains unanswered as to whether the dmPFC causally influences metacognitive bias and/or metacognitive sensitivity and efficiency.

In addition to the dmPFC’s involvement in monitoring task performance (Brown & Braver, 2005; Carter et al., 1998), dmPFC was ascribed a role for social cognition and mentalising (Christian et al., 2024; Konovalov et al., 2021; Majdandžić et al., 2016; Yao et al., 2021). Mentalising refers to the ability to understand the mental states of others and to appreciate that these may differ from our own (Kliemann & Adolphs, 2018). On a conceptual level, mentalising shares similarities with metacognition, involving both monitoring (monitoring the mental states of others) and control (using information about others’ mental states to predict their behaviour) (Frith, 2012). Therefore, Carruthers (2009) suggested metacognition to be a form of self-directed mentalising where humans turn their mindreading capacities on themselves. On the neural level, this is partially supported by an fMRI meta-analysis (Vaccaro & Fleming, 2018), reporting a slight overlap between metacognition and mentalising processes in the dmPFC, although metacognition and mentalising seem to be related to more posterior and anterior parts, respectively, of dmPFC. Results from another fMRI study suggest distinct, nonoverlapping neural representations of metacognition and mentalising (Jiang et al., 2022). There is thus no conclusive evidence that metacognition and mentalising are implemented by overlapping brain mechanisms. A further goal of our study was to assess whether the role of the dmPFC in metacognition can be explained by its involvement in mentalising.

Taken together, the current study tested the causal involvement of the dmPFC in metacognition and whether it can be explained by the dmPFC’s more general role for mentalising. We hypothesised that dmPFC tDCS would affect metacognitive (second-order) performance but not first-order task performance. Specifically, based on previous imaging research, we expected a (negative) stimulation effect on metacognitive bias (Ting et al., 2023), whereas we had no a priori predictions regarding the influence of tDCS on metacognitive sensitivity owing to the lack of conclusive previous results on a link between dmPFC activation and metacognitive sensitivity. We also predicted enhanced dmPFC excitability to enhance mentalising performance. To test our hypotheses, participants performed a perceptual decision task as a measure of metacognition and a false-belief task as a measure of mentalising while undergoing transcranial direct current stimulation (tDCS) to enhance dmPFC excitability. Together, this would provide a deeper understanding of the causal neural mechanisms underlying metacognition.

## Materials and methods

### Participants

The sample included a total of 35 participants (22 females, 33 right-handed, age range 19–29 years, *M* = 23.2, *SD* = 1.86). The sample size was determined with an a-priori power analysis ($\alpha$ = 0.05 two-tailed and power = 80%) based on the effect size of Cohen’s d = 0.54 observed in a previous tDCS study on metacognition (Soutschek et al., 2021), indicating a required minimum sample size of 29 participants. Participants had normal or corrected vision, confirmed the absence of a history of neurological or psychiatric disorders as well as any metal implants, and gave written informed consent before the experiment. They received a monetary compensation of 15€/h. The study was conducted in accordance with the ethical standards laid down in the Declaration of Helsinki (World Medical Association, 2013), and was approved by the ethics committee of the department for psychology at the Ludwig-Maximilians-Universität München (protocol number: “24_Soutschek_a”).

### Stimuli and task design

Participants performed two tasks while receiving tDCS stimulation: a mentalising task and a metacognition task. Both tasks were programmed in MATLAB, R2023a (www.mathworks.com) using Psychtoolbox (Brainard, 1997).

### Metacognition task

**Fig. 1:**
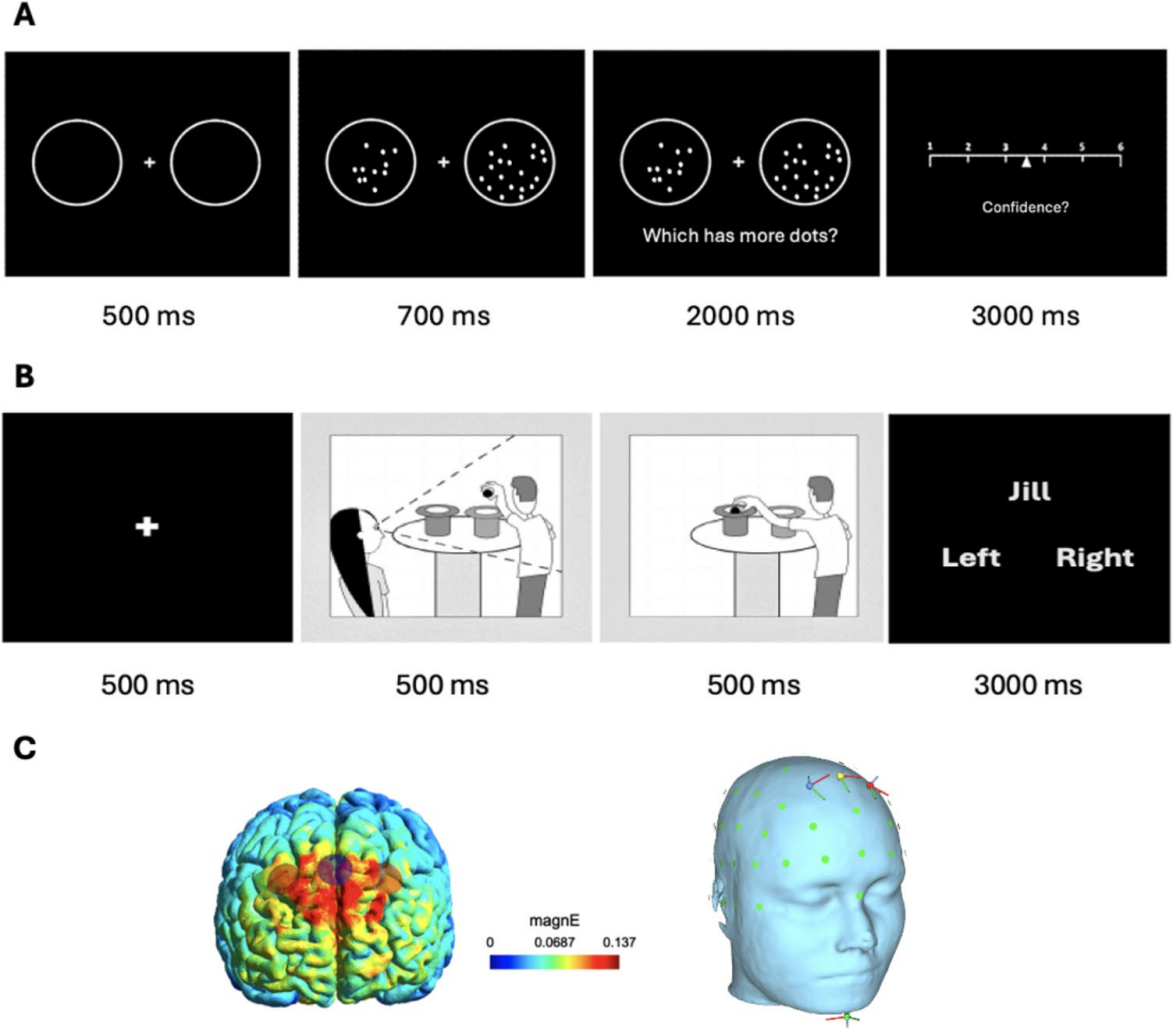
Trial sequence in both tasks. (**A**) In the metacognition task, retrieved and adapted from Fleming et al. (2014), participants viewed two circles containing various amounts of dots on a screen and had to decide which circle contained more dots. After each decision, they were asked to rate their confidence in their decision being correct. (**B**) The mentalising task, adapted from Dennis et al. (2012), constituted a false-belief-task that required participants to indicate their own or the observer Jill’s belief about the ball’s position in either one of two hats. In some conditions, Jill held a false belief, because the ball’s previous position had been switched in her absence (Jill-Switched condition). (**C**) Simulation of the tDCS stimulation (intensity = 1.5 mA), targeting the dmPFC using SimNIBS (Saturnino et al., 2019). Warmer colours indicate higher electric field density

Participants completed a perceptual task developed by Fleming et al. (2014) to measure metacognition in perceptual decisions. In each trial, participants were first shown two circles (diameter 5.1°), together with a fixation cross on the screen (500 ms), before they were presented with the same circles containing a variable, unequal number of dots (diameter 0.1°) for 700 ms. Participants were asked to indicate which of the circles contained the higher number of dots within 2000 ms by pressing the left or right arrow key on a standard keyboard for the left or right circle, respectively. The dot amount inside each circle was bound to 1–100, and the contrast between the two circles was adjusted to each individual’s performance, which was determined in a previous practice task using a one-up two-down staircase procedure (Fleming et al., 2014; García-Pérez, 1998). The purpose of the staircase procedure was to balance the difficulty of the task between individuals and thus to control for individual performance differences. After each choice, we presented participants with a sliding scale to indicate their confidence in having given the correct answer before (Fig. 1A). The scale ranged from 1 (low confidence) to 6 (high confidence), and participants had to select their level of subjective confidence within 3000 ms. By using the left and right arrow keys, participants could slide the cursor, which was initialised at a random location on each trial.

### Mentalising task

For the mentalising task, participants completed a modified pictorial false-belief task adapted from Dennis et al. (2012). Following a white fixation cross on the screen (500 ms), each trial consisted of a sequence of two images depicting a scene between two characters, Jack and Jill. In the first image, Jill observes Jack holding a ball over one of two hats (500 ms). In the second image, Jill is absent, and Jack drops the ball into either the same hat (condition: no switch) or into the other (condition: switch) (500 ms). The outcome comprises one of two possible conditions: either Jill’s belief about the current ball’s location is correct, or Jill’s belief about the ball’s location is incorrect (Fig. 1B). After the second image, we displayed a cue indicating whether participants should report the ball position from their own (“You”) or Jill’s perspective (“Jill”). Only the Jill-Switched condition, that is, when participants are required to report the ball position that diverges from the actual position they witnessed the ball to be in, includes a false belief scenario and requires mentalising processes to give the correct answer. In the Jill-No switch condition, participants again had to indicate the ball position from Jill’s perspective, but the ball position did not change between the images, such that there was no conflict between participants’ and Jill’s perspectives. Participants had 3000 ms to provide their answer whether they believed the ball to be in the left or right hat by pressing either the left or right arrow key, respectively.

### Procedure

After giving informed written consent, participants were presented with instructions and practice trials for both tasks, 10 trials for the mentalising and 60 trials for the metacognition task (which served also to calibrate the perceptual decision task to individual performance levels). Then, participants underwent the preparation of the tDCS set-up. The main experiment consisted of a total of 6 runs, two for the mentalising and four for the metacognition task (in counterbalanced order). Each run included two mini-blocks of the same task, one of which was performed under anodal tDCS, the other under sham tDCS. The order of anodal vs. sham mini-blocks was pseudorandomised and carried out unbeknownst to the participant. While the mentalising task mini-blocks consisted of 32 trials, a mini-block of the metacognition task included 24 trials. Both stimulation blocks had a duration of 2.5 min. The two mini-blocks were always separated by a break of 35 s to avoid after-effects of the previous stimulation (Christian et al., 2023; Moisa et al., 2016; Soutschek et al., 2021) while also allowing participants to rest.

### tDCS protocol

**Fig. 2:**
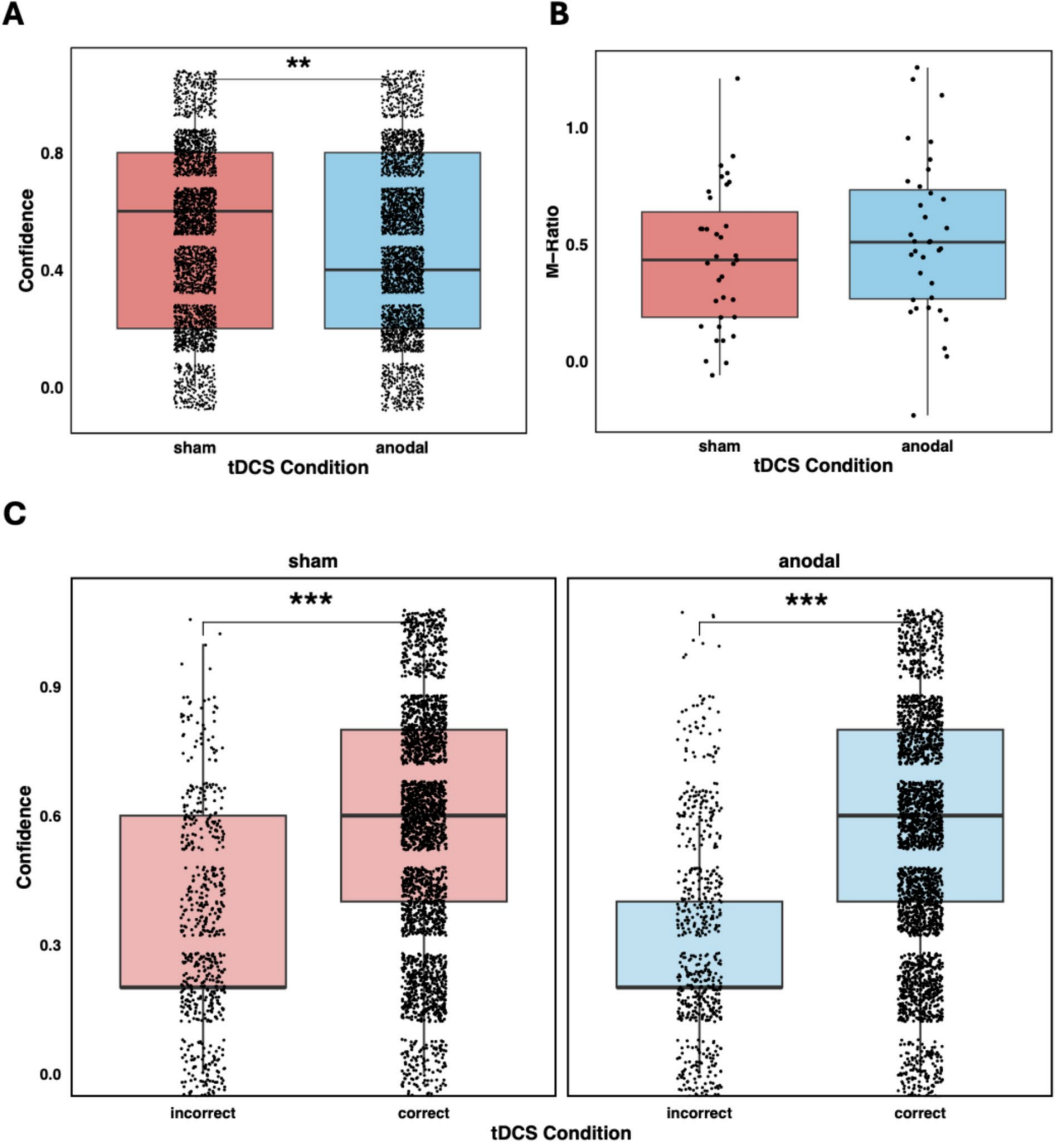
**(A)** Boxplot of confidence ratings in sham condition (red) vs. anodal tDCS condition (blue). Anodal tDCS significantly reduced confidence ratings compared with sham. (**B**) Boxplot of M-ratio values in the sham condition (red) and the anodal tDCS condition (blue). (**C**) Boxplots of confidence ratings for correct and incorrect responses under both anodal tDCS and sham. The analyses revealed no evidence for tDCS effects on metacognitive sensitivity or efficiency. Black dots represent individual data points

We applied anodal high-definition (HD) tDCS (Datta et al., 2009) using a 16-channel tDCS stimulator (neuroConn, Illmenau, Germany). Anodal stimulation increases cortical excitability by modulating neuronal resting membrane potentials, which increases the likelihood of depolarisation in the targeted area (Nitsche & Paulus, 2000). We applied an electrode set-up to target the dmPFC subregion (MNI coordinates x = −4, y = 40, z = 34) reported in the meta-analysis by Vaccaro & Fleming (2018) for the overlap between metacognition and mentalising. The active electrode was placed at position Fz according to the international 10–20 system and two reference electrodes equidistantly at positions F1 and F2. A third reference electrode was positioned right under the participant’s chin to induce a current flow deep into the brain (Soutschek et al., 2022). We used round rubber electrodes with a 2-cm diameter and attached them by using the Ten20 conductive paste (Ten20 EEG Conductive Paste, Weaver and Company). This HD-tDCS set-up with relatively small electrodes increases spatial precision compared with past tDCS research using larger electrodes, and the chin electrode allowed for focused stimulation of deeper dmPFC subregions that were linked to metacognition by Vaccaro & Fleming (2018) and that could not be reached by alternative methods, such as transcranial magnetic stimulation. To prevent any movement during the session, we fixated the electrodes with gauze wrapped around the participant’s head. In the anodal tDCS condition, we applied tDCS with an intensity of 1.5 mA at the active Fz electrode and –0.5 mA at each of the three reference electrodes. We note that current intensities above 1.5 mA may lead to weaker effects on cognition due to the hypothesised inverted u-shaped dose-response curve for tDCS (Ehrhardt et al., 2021). In the sham condition, the same current strength was administered for the first 15 s of the mini-block before turning the stimulation off during the rest of the mini-block. The same was done for another 10 s after 1 min during the mini-block. Before the experiment, electric field simulations were performed using the SimNIBS 2.1 toolbox (Saturnino et al., 2019) to ensure the target area was stimulated (Fig. 2C). The simulations suggest a field strength of 0.09 V/m in a region-of-interest (diameter = 10 mm) around the peak coordinates reported in Vaccaro & Fleming (2018).

## Statistical analysis

We analysed data with generalised mixed linear models (GLMMs) in R (R Core Team, 2023) using the packages *lme4* (Bates et al., 2015) and *lmerTest* (Kuznetsova et al., 2017). In all analyses, the alpha threshold was set to 5% two-tailed. Furthermore, we computed Bayes factors (BF) by comparing the Akaike Information Criteria (AIC, Akaike, 1974) of the GLMMs with a null model, following previous procedures (Raftery, 1995; Wagenmakers, 2007). For all BFs, we report BF_10_ to quantify the evidence in favour of the alternative hypothesis over the null hypothesis. Trials where participants had not reacted were excluded from the analysis. This affected 75 trials in the metacognition task (1.1% of all trials), and 6 trials in the mentalising task (0.13% of all trials). We checked for staircase convergence by ensuring that all participants exhibited above-chance level performance (accuracy was above 50% for all participants, mean accuracy = 82%). There was also sufficient variation in participants’ second-order confidence ratings (i.e., all participants responded with at least three different values on the confidence scale).

In the metacognition task, we analysed first-level perceptual performance with separate GLMMs on reaction times (RT) and accuracy, respectively. In these GLMMs, we regressed log-transformed RTs or accuracy (0 = incorrect, 1 = correct) on a dummy-coded fixed effect predictor for tDCS (0 = sham, 1 = anodal) and on a continuous predictor for task difficulty measured by the contrast in dot amounts between the two circles (lower contrast values indicate higher task difficulty). Both predictors were also modelled as random slope in addition to participant-specific random intercepts.

In addition to first-order task performance, we tested for stimulation effects on metacognitive bias and sensitivity. For metacognitive bias, we regressed confidence ratings on tDCS as both fixed effect and random slope in addition to random intercepts. We also computed a GLMM additionally, including RT and accuracy as fixed-effect and random-effect predictors to control for the influence of these variables on confidence ratings. Following previous studies (De Martino et al., 2013; Fleming et al., 2014; Soutschek et al., 2021), we define metacognitive sensitivity as the strength of the relationship between accuracy (objective task performance) and subjective confidence. In our analysis, we assessed tDCS effects on metacognitive sensitivity by computing a GLMM where the dependent variable confidence was predicted by tDCS, Accuracy, and their interaction as fixed effects as well as participant-specific random intercepts and random slopes for all fixed effects. Additionally, in an analysis of both metacognitive sensitivity and efficiency, we computed Meta-d’ and M-ratio to quantify participants’ ability to discriminate between correct and incorrect responses in their confidence ratings while simultaneously controlling for differences in task performance and metacognitive bias (Fleming & Lau, 2014). To this end, we computed participants’ Meta-d’ and M-ratio scores separately for sham and anodal tDCS using the *metaSDT*-package (Craddock, 2021). In addition, we calculated metacognitive noise as a measure of the variability in the confidence criterion (Shekhar & Rahnev, 2021), separately for each participant and tDCS condition following the computational approach described by Rahnev (2023). The MATLAB scripts for computing metacognitive noise are available on GitHub (https://osf.io/y5w2d/). We ran paired *t*-tests to compare individual Meta-d’, M-ratio, as well as metacognitive noise scores between anodal and sham tDCS.

For the mentalising task, we regressed both log-transformed RTs and accuracy (0 = incorrect, 1 = correct) in two separate models on fixed-effect predictors for tDCS, Perspective (1 = Self, 2 = Jill), Switch (1 = No Switch, 2 = Switch) and all interaction effects. All fixed effects were also modelled as random slopes in addition to random intercepts to account for between-subject variability. For the RT analysis, we only used correct trials.

Lastly, to assess the relationship between mentalising and metacognition, we calculated the correlations between participants’ performance in the two tasks under sham. For this, we used participants’ mean values across both tasks: For mentalising performance, we computed the log-RT difference for the interaction between perspective and switch ((RT_switch_ – RT_no switch_)_Jill_ – (RT_switch_ – RT_no switch_)_Self_), with values close to or below zero constituting high mentalising skills and higher values implying weaker mentalising abilities. As measures of metacognitive bias and sensitivity, we used participants’ mean confidence and M-ratio scores, respectively. Furthermore, we examined how the changes between anodal versus sham tDCS in metacognition and mentalising performance correlate with each other. For this, we calculated the log-RT difference for the Perspective × Switch interaction between anodal and sham tDCS for the mentalising task (((RT_switch_ – RT_no switch_)_Jill_ – (RT_switch_ – RT_no switch_)_Self_)_sham_ – ((RT_switch_ – RT_no switch_)_Jill_ – (RT_switch_ – RT_no switch_)_Self_)_anodal_). For the metacognition task, we calculated mean confidence differences between anodal and sham tDCS ((mean confidence)_sham_ – (mean confidence)_anodal_), as well as M-ratio differences between the two tDCS conditions ((M-ratio)_sham_ – (M-ratio)_anodal_).

## Results

### dmPFC tDCS causally affects metacognitive bias

We first ensured that tDCS did not affect participants’ first-order perceptual performance in the metacognition task. We observed no significant effect of tDCS on log-transformed RTs, ${\upchi }^{2}$(1) = 0.99, ß = 0.02, *t*(41) = 1.00, *p* = .33, BF_10_ < 0.001, or on accuracy, ${\upchi }^{2}$(1) = 0.60, ß = 0.15, *z* = 0.77, *p* = .44, BF_10_ < 0.001, when controlling for difficulty (contrast). For both models we found a significant effect of difficulty (contrast), ${\upchi }^{2}$(1) = 5.70, ß = –0.01, *t*(34) = 2.39, *p* = .02, BF_10_ = 3.44x10^15^ for RTs, and ${\upchi }^{2}$(1) = 91.79, ß = 0.25, *z* = 9.58, *p* < .001, BF_10_ = 2.34x10^57^ for accuracy. We found no significant interaction between tDCS and difficulty, neither for the RT model, ${\upchi }^{2}$(1) = 0.10, ß = –0.001, *t*(55) = 0.32, *p* = .75, BF_10_ < 0.001, nor the accuracy model, ${\upchi }^{2}$(1) = 0.61, ß = –0.02, *z* = 0.78, *p* = .43, BF_10_ = 0.01. There was thus no evidence for tDCS effects on first-order perceptual decision making.

Next, we tested the hypothesized causal contribution of dmPFC to metacognition. First, regarding metacognitive bias, we observed a significant negative effect of tDCS on confidence, ${\upchi }^{2}$(1) = 9.65, ß = –0.02, *t*(34) = 3.11, *p* = .004, BF_10_ = 2.52, suggesting that increasing dmPFC excitability decreased subjective confidence in perceptual decisions (Fig. 2A). This main effect of tDCS was robust to adding RTs and accuracy as control variables, *p* = .02. This provides evidence for a causal influence of dmPFC activation on metacognitive bias. Then, we tested the influence of dmPFC tDCS on metacognitive efficiency. Model-based comparisons of M-ratio (metacognitive efficiency) values revealed no significant difference between sham (*M =* 0.44, *SD =* 0.30) and anodal tDCS (*M =* 0.53, *SD =* 0.34), *t*(34) = 1.32, *p* = .20 (Fig. 2B), neither did we find a significant difference between Meta-d’ values (metacognitive sensitivity) in the sham (*M =* 0.63, *SD =* 0.41) and anodal tDCS condition (*M =* 0.73, *SD =* 0.48), *t*(34) = –1.25, *p* = .22. Moreover, the comparison of metacognitive noise as measure of the variability in the confidence criterion between sham (*M* = 0.21, *SD* = 0.25) and anodal tDCS (*M* = 0.43, *SD* = 0.57) showed only a nonsignificant trend-level difference, *t*(34) = 2.00, *p* = .053. This result therefore should be interpreted with caution. Further analyses regressing subjective confidence on objective performance accuracy and tDCS revealed that overall, participants possessed metacognitive access to the accuracy of their decisions, as indicated by a significant main effect of Accuracy, ${\upchi }^{2}$(1) = 214.67, ß = 0.83, *t*(38*)* = 14.65, *p* < .001, BF_10_ = 3.39x10^124^. However, the analysis revealed no significant main effect of tDCS, ${\upchi }^{2}$(1) = 1.29, ß = 0.06, *t*(3027) = 1.14 *p* = .26, BF_10_ < 0.001, or a significant tDCS × Accuracy interaction, ${\upchi }^{2}$(1) = 1.57, ß = –0.08, *t*(2477) = 1.25, *p* = .21, BF_10_ < 0.01 (Fig. 2C). Contrary to metacognitive bias, there was no evidence for tDCS effects on metacognitive efficiency or metacognitive sensitivity.

#### No evidence for effects of dmPFC tDCS on mentalising

**Fig. 3:**
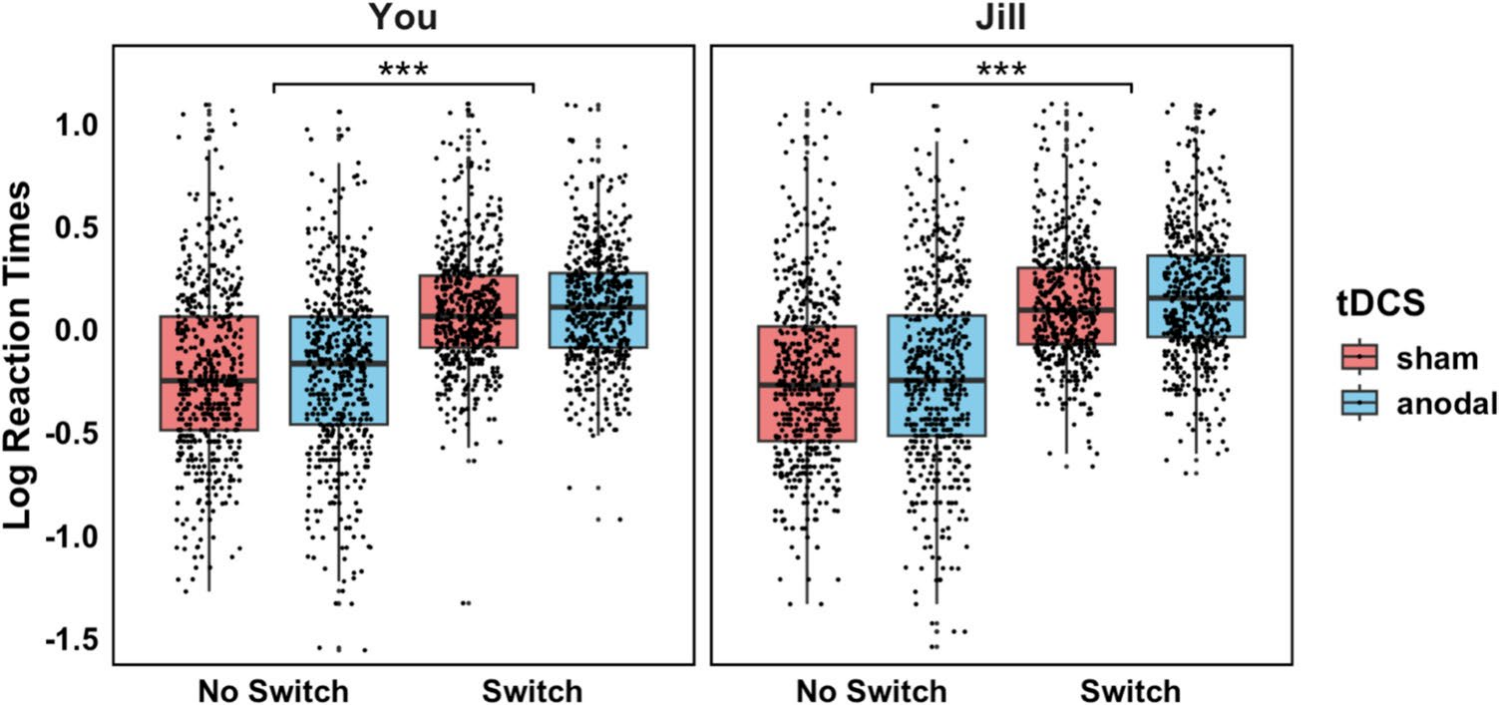
Boxplots of mean log-transformed RTs in the mentalising task in the sham (**red**) vs. anodal tDCS condition (**blue**) as a function of Perspective condition (You vs. Jill) and Switch condition (No Switch vs. Switch). There was no evidence for significant tDCS effects on mentalising

In the mentalising task, RTs were slower for Switch compared with No Switch trials, ${\upchi }^{2}$(1) = 94.13, ß = 0.31, *t*(35) = 9.70, *p <* .001, BF_10_ = 4.11x10^273^. We also observed a tendency towards a Perspective × Switch interaction, ${\upchi }^{2}$(1) = 3.66, ß = 0.06, *t*(44) = 1.91, *p* = .06, BF_10_ < 0.001, in line with the assumption that switch trials are more difficult than nonswitch trials in the Jill compared with the Self-condition due to mentalising demands $.$ Contrary to our hypothesis, we found no significant effects of tDCS, all ${\upchi }^{2}$ < 0.42, all *t* < 0.65, all *p* > .52, BF_10_ < 0.001 (Fig. 3). Also, a separate GLMM on accuracy in mentalising performance revealed no evidence for significant tDCS effects, all ${\upchi }^{2}$ < 0.43, all *z* < 0.65, all *p* > .52, BF_10_ < 0.001. The current findings thus provide no support for dmPFC involvement in mentalising.

**Fig. 4:**
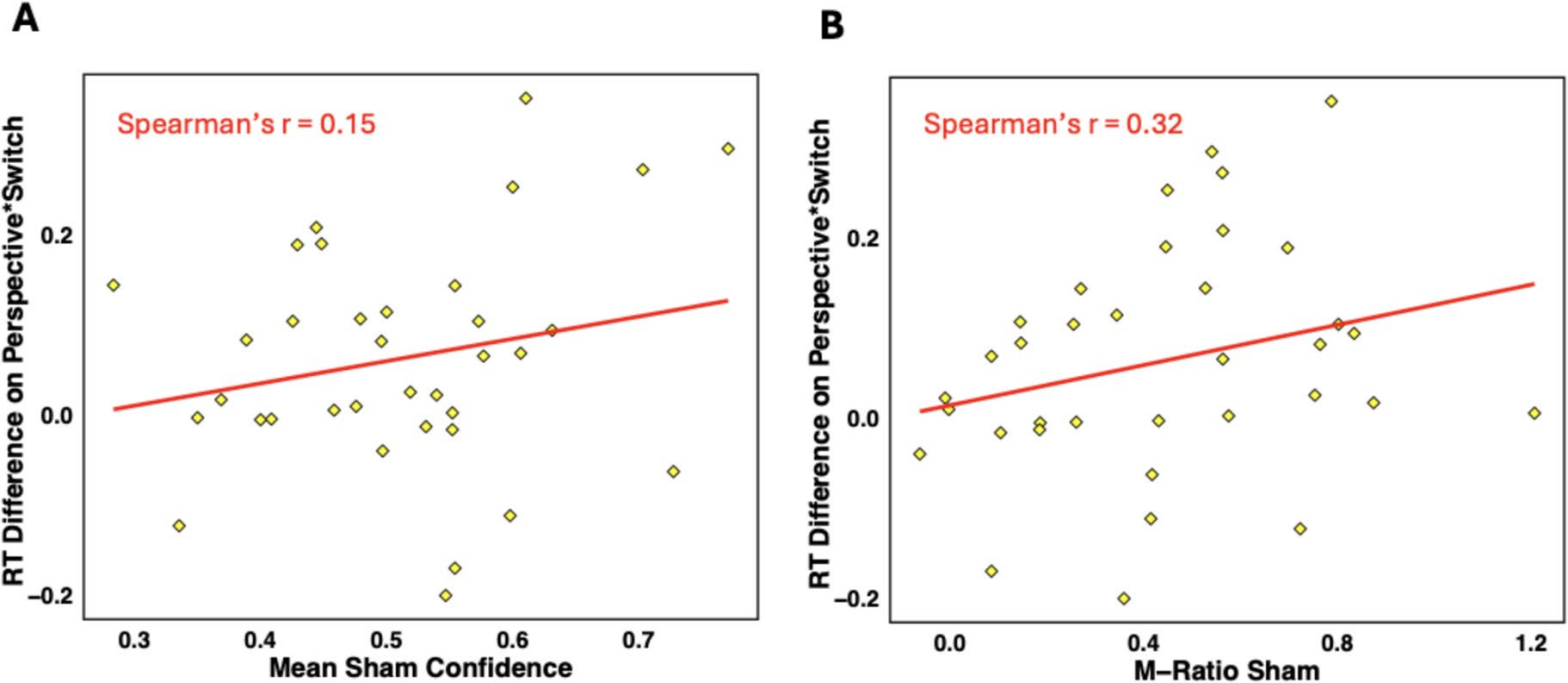
Scatterplots of reaction time difference in the Perspective × Switch interaction with mean sham confidence values (**A**), and sham M-ratio values (**B**). Interaction values close to zero constitute high mentalising skills whereas higher values signify poorer mentalising abilities

Lastly, based on theoretical accounts of metacognition as self-directed mentalising, we tested for the hypothesised link between metacognition and mentalising under sham. We found no significant correlation between mentalising (Perspective × Switch interaction in log-RTs) and mean confidence as measure of metacognitive bias, Spearman’s *r* = 0.15, *p* = .40, BF_10_ = 0.73 (Fig. 4A). However, there was a positive trend-level correlation between RT difference and M-ratio scores, Spearman’s *r* = 0.32, *p* = .06, BF_10_ = 1.02 (Fig. 4B), suggesting that—if anything—lower RT differences, which are indicative of better mentalising, are associated with lower M-ratio. When we tested for correlations between tDCS-induced performance changes, we observed no significant correlations between tDCS effects on mentalising performance and tDCS effects on mean confidence, Spearman’s *r* = –0.027, *p* = .88, BF_10_ = 0.38, nor in M-ratio, Spearman’s *r* = –0.05, *p* = .76, BF_10_ = 0.65. Thus, the current data do not offer empirical evidence for the theory of metacognition as a self-directed form of mentalising.

## Discussion

Conceptually and empirically, metacognition has been linked to dmPFC activation (Ting et al., 2023; Vaccaro & Fleming, 2018); however, previous investigations were only correlative and did not distinguish between metacognitive bias and sensitivity. Here, we show that anodal tDCS targeting the dmPFC significantly decreases subjective confidence ratings, providing evidence for a causal role of dmPFC in encoding metacognitive bias. In contrast, we found no significant effect of dmPFC tDCS on metacognitive sensitivity, efficiency, noise, or on first-order task performance in the perceptual decision task. Taken together, this suggests a role of dmPFC for representing specifically metacognitive bias.

The negative effect of dmPFC tDCS on confidence ties together with recent evidence of a negative correlation between reported confidence and dmPFC activity in fMRI (Ting et al., 2023). In the light of the strong dmPFC tDCS effect on confidence, the lack of a similar effect on metacognitive sensitivity and efficiency implies at least partially distinct neural mechanisms to underlie metacognitive bias on the one hand, and accuracy and efficiency on the other. It should be noted that, given the trend-level effects of dmPFC tDCS on metacognitive noise, it remains possible that the null effects on first-order perceptual decisions and on metacognitive sensitivity or efficiency can be explained by (nonsignificant) tDCS-induced changes in metacognitive noise (Zheng et al., 2024).

In a meta-analysis, Vaccaro & Fleming (2018) reported an association of confidence levels (as indicator of metacognitive bias) with a domain-general network including the dmPFC, dlPFC, and ventromedial PFC. Metacognitive sensitivity, in contrast, was found to be causally implemented by FPC (Shekhar & Rahnev, 2018; Soutschek et al., 2022), whereas FPC stimulation showed no effects on self-reported confidence (i.e., metacognitive bias). Brain stimulation studies targeting the dlPFC observed selective effects on metacognitive bias (Bona & Silvanto, 2014; Xue et al., 2023), although other studies reported also stimulation effects on metacognitive sensitivity (Chua et al., 2017; Gaynor & Chua, 2019; Rounis et al., 2010, for a comprehensive review, see Saccenti et al., 2024). This seems to indicate that the neural mechanisms underlying metacognitive bias and sensitivity may, in fact, involve dissociable neural mechanisms. Presumably, metacognitive sensitivity and efficiency require the brain to integrate different information to assess and compare information about first-order task performance and confidence (represented in dmPFC and dlPFC). This integration process is implemented by anterior parts of PFC, which are at the top of a hierarchy of prefrontal control processes (Koechlin & Hyafil, 2007; Koechlin & Summerfield, 2007).

The dmPFC is widely established to be associated with flexible, adaptive control of behaviour, including error monitoring (Brown & Braver, 2005; Carter et al., 1998), conflict monitoring (Barch et al., 2000; Botvinick et al., 1999; Kerns et al., 2004), and decision-making under uncertainty (Hadland et al., 2003; Kennerley et al., 2006; Rushworth et al., 2004). Several theories have suggested the dmPFC to detect situations characterised by high objective uncertainty (e.g., high task difficulty or prediction errors) (Botvinick et al., 1999; Fan, 2014), which triggers increased cognitive control processes or updates of mental representations (Alexander & Brown, 2015; Brown & Braver, 2005). In fact, activity in this region increases in situations requiring mental updating (Kolling et al., 2016). Until now, however, studies have focused mostly on the dmPFC’s connection with *objective* uncertainty and neglected the question whether this applies to *subjective* uncertainty as well. Uncertainty results from mental representations of the environment not aligning with our expectations, so by updating our beliefs about our world, we try to reduce uncertainty (Clairis & Lopez-Perem, 2023). The observed causal influence of the dmPFC on subjective, self-reported uncertainty may connect metacognition with research on conflict monitoring and cognitive control adjustments, deepening our understanding of the dmPFC’s role in flexible, adaptive behaviour.

In contrast to metacognition, we observed no significant effect of dmPFC tDCS on performance in the mentalising task, providing no support for the proposed overlap between mentalising and metacognition in the dmPFC. Vaccaro & Fleming (2018) reported a weak overlap between mentalising and metacognition in the anterior dmPFC, although metacognition was found to be more strongly associated with posterior parts of dmPFC, whereas unique activations for mentalising were observed in anterior dmPFC, the temporo-parietal junction, and temporal regions. This is in line with other stimulation studies that found significant effects on mentalising in more anterior parts of the dmPFC than the current study (Martin et al., 2017, 2019, for a comprehensive overview, see the meta-analysis by Yao et al., 2021) and the fMRI study by Jiang et al. (2022), reporting a delineation between the respective neural underpinnings of metacognition and mentalising.

At variance with the assumption that metacognition represents a form of self-directed mentalising, we observed no correlation of mentalising with metacognitive bias and only a trend-level negative, rather than positive, correlation with metacognitive sensitivity. The observed association of mentalising with metacognitive sensitivity rather than bias is consistent with the neural findings showing dmPFC tDCS effects only on metacognitive bias, not on mentalising or metacognitive sensitivity or efficiency. The direction of this trend-level correlation suggests that better metacognitive skills are associated with lower mentalising performance, contrary to the theory of metacognition as a self-directed form of mentalising.

This finding should be interpreted with caution owing to limitations of the current investigation. First, because the administered metacognitive task measured perceptual metacognition, it allows drawing inferences about metacognitive processes in perceptual decisions only. There is an ongoing debate in the field of metacognition about whether metacognitive processes can be regarded as domain general or domain specific. Some studies report correlations across different metacognitive tasks and modalities (Ais et al., 2016; Faivre et al., 2018; McCurdy et al., 2013), whereas others found no significant correlations (Baird et al., 2013; Fitzgerald et al., 2017; Garfinkel et al., 2016). Given this ongoing debate, mentalising might correlate with other, presumably more social metacognitive domains than the one we examined. As a second limitation, the mentalising task we used—despite being well established (Dennis et al., 2012; Mossad et al., 2016)—might not have been sensitive enough to brain stimulation effects or individual difference analyses. Thus, while the results of our current study provide no evidence for accounts of metacognition as self-directed mentalising, future studies may need to validate our findings by using other mentalising and metacognition tasks. Third, because we did not collect data on participants’ subjective physical sensations during the stimulation, we cannot draw inferences about their blindness to the different tDCS conditions. While previous studies found that participants are not able to discern between active and sham conditions of tDCS above chance-level for low intensities, such as used in the current studies (Tang et al., 2016), we cannot formally rule out potential site-unspecific stimulation effects on behaviour.

## Conclusions

We show that enhancing dmPFC excitability decreased subjective confidence reports, providing evidence for a causal involvement of the dmPFC in metacognitive bias. Interestingly, no effect was observed for metacognitive sensitivity, suggesting dissociable neural substrates to underlie different aspects of metacognition. Furthermore, we found no effect of dmPFC tDCS on mentalising, providing no evidence for a common role of dmPFC for perceptual metacognition and mentalising. These findings deepen our understanding of the causal neural mechanisms underlying different subfacets of metacognition as well as of the relationship between metacognition and mentalising.

## Data Availability

The data supporting the conclusions of the current study will be made publicly available on the Open Science Framework (OSF).

## Code availability

The code that was used for data analysis is available upon request.
